# Alarming findings of psycho-socio-spiritual interventions on physical, mental, and social health for children with cancer and their families in low- and middle-income countries: a meta-analysis

**DOI:** 10.3389/fpsyt.2025.1533599

**Published:** 2025-04-28

**Authors:** Hisham Morsi, Tooba Nadeem Akhtar, Harriet Dean Miller, Özge Balkaya, Jeanine Clapsaddle, Holly Clark

**Affiliations:** ^1^ Child Life Services, Pediatric Potential Inc, Minneapolis, MN, United States; ^2^ Plymouth, Lead of Quality of Life and Transitional Care, Clinical Advancement Department, Hamad Medical Corporation, Doha, Qatar; ^3^ Department of Psychology, Honorary Kingston University, London, United Kingdom; ^4^ Trinity Centre for Global Health, Trinity College Dublin, Dublin, Ireland; ^5^ Institute of Child Development, University of Minnesota, Minneapolis, MN, United States

**Keywords:** childhood neoplasms, psychosocial care, psycho-oncology, quality of life, wellbeing

## Abstract

**Background:**

Children residing in low- and middle-income countries (LMICs) are at a higher risk of cancer. The provision of psycho-socio-spiritual care to address stressors accompanying a cancer diagnosis is largely unknown in these countries, and evidence on psycho-socio-spiritual interventions in LMICs remains unexplored.

**Objective:**

This meta-analysis aimed to synthesize findings on psycho-socio-spiritual interventions for children and families with cancer in LMICs in comparison with those from resource-rich developing nations.

**Design:**

This study employs a prospective comprehensive meta-analysis approach.

**Setting(s):**

The study covers low- and middle-income countries and resource-rich developing countries as per the World Bank classification.

**Participants:**

The participants came from a total of 18 studies that recruited 3,072 children (0–18) with cancer or their family members and carers who were included in this meta-analysis.

**Methods:**

A systematic search of five databases PubMed, PsycINFO, CINAHL, Cochrane Databases, and a gray literature ProQuest was conducted to identify all possible hits. Following screening, data were extracted on a comprehensive list of variables to allow pooled and moderation analysis. The meta-analysis was performed via CMA-v2, the quality of the included studies was assessed via the Cochrane software “Risk of Bias-v2.0 (RoB2)”, and PRISMA and AMSTAR 2 guidelines were followed throughout.

**Results:**

A highly significant OR of 4.933 (95% CI: 3.423–7.108, *p <* 0.0001) indicated approximately fivefold improvement in children and families with cancer in 11 LMICs as a result of the psycho-socio-spiritual interventions. Four more LMICs showed evidence of qualitative psycho-socio-spiritual services for children with cancer. The quality of evidence was rated as 2B in most of the eligible studies. We established a model that can test >400,000 combinations of factors.

**Conclusions:**

The childhood oncology community has been alerted on the lack of equitable holistic care for children and families with cancer in 126 out of the 137 LMICs and to seize the opportunity to target the underserved populations through development, adaptation, and investment in psycho-socio-spiritual care. Our model can aid in future studies and policy making.

**Systematic review registration:**

https://www.crd.york.ac.uk/prospero/, identifier CRD42023460114.

## 1 Introduction

Children residing in low- and middle-income countries (LMICs) are at a higher risk of cancer, where approximately 80% of children with cancer reside, and those without access to management do not survive. In contrast, >75% of children diagnosed with cancer in resource-rich countries survive for 5 years and may live a full lifetime (1).

The risk factors for developing cancer in LMICs include chronic infections and air pollutants (2) and result in mortality that is three times higher than those in resource-rich countries (3, 4) with specific malignancies having the highest mortality-to-incidence rates compared to high-income countries (5).

For those experiencing draining cancer treatment, psychosocial care is an essential component that ensures a quality-adjusted survival (6).

The provision of high-quality psycho-socio-spiritual care has not matched the global pace of advancing biomedical care (7). In further contrast with patients in resource-rich USA and Europe, the provision of medical care itself is poorer (4) as are psycho-socio-spiritual interventions for children with cancer. The focus of survival-oriented healthcare may remain attentive to immediate medical treatments rather than a comprehensive care that simultaneously addresses wellbeing (8–10).

Focused psycho-socio-spiritual interventions for traumatic stressors in children in LMICs were systematically reviewed, and it was identified that only 16 LMICs out of the 137 countries on the World Bank list applied a psycho-socio-spiritual intervention (9, 11).

### 1.1 Psychosocial impacts of a cancer diagnosis

Chida et al., in their exploration, found that psychosocial stressors are associated with a higher cancer incidence, poorer cancer survival, and higher cancer mortality that could be mediated through behavioral pathways, such as lifestyle choices or through the activation and over-activation of the hypothalamic–pituitary–adrenal axis, activation of oncogenic viruses, and impaired immuno-surveillance (12).

Similarly, Lee et al. reported that children with cancer were 1.57 times more likely to develop severe symptoms of depression, 1.29 times more likely to develop anxiety, 1.56 times more likely to develop psychotic disorders, and have a higher risk of suicide mortality compared to the general population. They highlighted the need for early identification and management strategies to prevent and mitigate the psycho-socio-spiritual consequences of cancer care for children (13).

### 1.2 Evidence-based psychosocial interventions in resource-rich settings

Even with psychosocial interventions in resource-rich developed countries, Van Der Kurk et al. reported that patients and survivors of cancer in rural areas experience higher levels of psycho-socio-spiritual morbidity, have unmet needs compared to urban counterparts, experience unique challenges of limited access to services, social isolation, and financial and logistical burdens, and demonstrate poorer social status, emotional health, and quality of life (14).

Psycho-social-spiritual interventions are instrumental in addressing such challenges as they encompass many therapeutic modalities that utilize non-pharmacological, sensory-based techniques to mitigate the impact of medical interventions. Therapeutic and normative developmental play, procedural preparation, education and support, bibliotherapy, art therapy, music therapy, yoga, dance and movement therapies, prayer, meditation, hypnosis, biofeedback, and guided imagery are the most prevalent interventions for pediatric patients. Within westernized medical communities, these interventions are widely available to pediatric patients as developmentally and medically appropriate. However, the provision of psycho-social-spiritual interventions in LMIC countries, as identified by this metanalysis, is limited.

Psycho-social-spiritual interventions demonstrate benefits to pediatric patients across the age spectrum. Music therapy techniques have been shown to reduce pain in infants undergoing injections and venipuncture (15), promote neural development in pre-term infants, and reduce parental stress in neonatal units (16, 17). Reading and bibliotherapy techniques have been shown to support language development in infants in the NICU (18) as well as children experiencing trauma (19, 20). As children age, techniques such as therapeutic play and parental comforting facilitate coping in hospitalized children (20, 21), while art therapy promotes communication and emotional regulation for hospitalized children (22).

Spirituality is a fundamental dimension of human health and a source of strength, motivation, and coping with dire diseases such as childhood cancers. Spiritual interventions boost the quality of life (QoL) of the family and child which is a treatment outcome endpoint of all management protocols. Spiritual care complements the physical and psycho-social interventions, acknowledges the spiritual aspect of a whole human being, and targets the spiritual root causes of disease (23). In this meta-analysis, spiritual interventions included writing prayers, counselling by a religious advisor on life, death, and divine fate, moral boosting, rituals, and recitation of holy text and prayers during pilgrimage. Other interventions include meditation, group rituals, and engaging with faith-based groups (23–25). They foster a sense of being, belonging, hope, connectedness, resilience, psycho-social coping, reduced stress, emotional intelligence, and even an enhanced immune system (26, 27).

However, the integration of spiritual interventions remains underexplored globally, and with limited resources in low- and middle-income countries (LMICs), the priority is given to physical care even at the expense of holistic care (24, 26, 27). In our letter to the editor, we highlighted the critical need to investigate the role and impact of spiritual interventions on the holistic wellbeing of patients with cancer in randomized controlled trials that represent the gold standard in medical evidence (24).

Although many psycho-social-spiritual interventions, such as yoga and guided imagery, can be done without additional physical resources, many of these interventions require materials such as toys, books, and paint to be efficacious. As such, LMICs with limited financial resources may be challenged to implement these services for patients and families.

### 1.3 Relevance of the current meta-analysis

We were unable to find meta-analyses that studied the impact of psycho-socio-spiritual interventions on children with cancer in LMICs. While a recent scoping review was carried out by Cabanes et al. on supportive care for cancer in LMICs, it did not review the impact of psycho-socio-spiritual care (28). McCutchan and colleagues studied the psychosocial impact of a limited area of help-seeking behavior in adults with cancer in LMICs but did not include children (29). Moreover, the only recent systematic review of psychosocial interventions in LMICs included adult patients with dementia and not children or cancer diagnoses (30). Seah et al. reviewed more than 50,000 publications for the magnitude of treatment abandonment for children with CNS tumors but did not study the impact of psychosocial interventions on abandonment rates or treatment outcome (31). The most recent systematic reviews in LMICs were observational with no quantification of impact of interventions on outcome (32, 33).

Furthermore, Kruk et al. underscored the inadequate state of psychosocial care for children, adolescents, and young adults in LMICs, noting that despite improvements in health outcomes over the past three decades, significant challenges remain and notably affect the services for mental health, trauma, and chronic conditions. They advocated for essential reforms, the integration of high-quality services into existing social frameworks, and a focus on unmet health needs, steps that are crucial for enhancing the effectiveness of wellbeing programs and improving the developmental health of children in LMICs (34).

### 1.4 Aims of the current systematic review and meta-analysis

This meta-analysis aims to promote the development of psycho-social-spiritual interventions for underserved children and families with cancer in LMICs through the demonstration of service deficits when compared with resource-rich developing nations. To align with the Populations, Interventions, Comparators, Outcomes, and Study designs (PICOS) framework, we focused on children with cancer and their families in LMICs who received psycho-socio-spiritual interventions. Our primary objective was to determine the efficacy of these interventions in improving the quality of life of children with cancer and their families while employing resource-rich developing countries for comparison rather than resourceful developed countries.

1. Are psycho-socio-spiritual interventions effective in improving outcomes for children with cancer and their families/caregivers in LMICs?
2. How diverse is the impact of psycho-socio-spiritual intervention on the wellbeing of children with cancer and their families?
3. How is the impact of psycho-socio-spiritual care assessed in LMICs?

## 2 Methods

To identify all relevant publications on psycho-socio-spiritual interventions for children with cancer, a literature review was conducted in accordance with the PRISMA guidelines. The study was registered on the Prospero register and followed a pre-registered protocol (35), and we explored a wide list of moderators (**Table 1**).

**Table 1:** List of all twenty covariates that were coded as moderators, the 1^st^ ten of which were incorporated in the heterogeneity meta-regression analysis.

Covariate	Type	Values and Direction of model				
Quality of study design	Continuous	1 – 8	Low	High	
Number of sessions	Continuous	1 – 12	Less	More	
Duration % hours	Continuous	0.1 – 20.56%	Shorter	Longer	
Type of Disease	Categorical	Cancer	Acute lymphocytic leukemia		
Reported outcome	Categorical	Single	Multiple		
Allocation	Categorical	Allocated	Quasi	Convenience	Random
Intervention	Categorical	Passive Support	Active Participation		
Risk of bias	Categorical	Concern	No risk		
Target	Categorical	Patient	Family		
Medical reform in the country during previous 20 years	Categorical	No	Yes		
Other covariates that were not included in the meta-regression model					
Countries	Sample size		Total hours	Gender	Age	
Category of intervention	Year of publication		Delivery setting	Assessment tool	Provider involved

The quality of study was scored as a continuous variable (Score of 1 for retrospective, 2 for semiexperimental, 3 for quazi pre and post, 4 for cross sectionals randomized, 5 for convenience sampling, 6 for clinical trial, 7 for randomized controlled trial and 8 for longitudinal randomized controlled trial) and the duration was standardized as the % of hours to the total hours for all studies. The highlighted cells represent the value of the variable that was associated with a favorable outcome. The different possible combinations of the moderators in the meta-regression-based simulation were 491,520.

### 2.1 Search strategy

A comprehensive search for literature that developed, adapted, or evaluated psycho-socio-spiritual or psychologically informed interventions for children with cancer and/or their caregivers in LMICs and resource-rich developing countries was carried out in December 2023 across four databases—PubMed, PsycINFO, CINAHL, and Cochrane—for published work and ProQuest for gray literature. The search was only restricted to the title field. Screening followed an eligibility criterion to include literature published in the last 13 years following a recommended 10–15 years of restriction reported to be optimal for speed under a tolerable accuracy of 15% (36, 37).

A review of existing literature was conducted to find suitable search terms related to psycho-socio-spiritual interventions and LMICs or resource-rich developing nations. These terms were then combined and linked using Boolean operators for the search queries (**Supplementary Material Appendix A**).

#### 2.1.1 Inclusion criteria

Publications were included if they were psycho-socio-spiritual interventions, were provided to children (0 to 18 years) with/or survived cancer, their parents, siblings, or caregivers, involved children under treatment for at least 6 months or have received at least two sessions of chemotherapy, were provided in LMICs or resource-rich developing nation, took place in multi-center or mixed-resource settings, targeted symptom alleviation or prevention and psycho-socio-spiritual enhancement, published as case reports, clinical trials, observational studies, theses, and carried out during the last 13 years.

#### 2.1.2 Exclusion criteria

Studies were excluded from the meta-analysis if they did not report an effect size, were limited to healthcare provider perspectives or feedback only, were provided in a high-income developed nation setting, not indexed as a psycho-socio-spiritual or psychologically informed intervention, or targeted participants over the age of 18 years to retain our focus on children exclusively and maintain a homogeneity in developmental stages.

While we believe that healthcare provider perspectives contribute to care outcomes, it is possible that their responses or feedback are influenced by their vested interests in the organization that they serve and other factors that may lead to our study lacking the depth and outcomes that we sought.

We included all cross-sectional, longitudinal, and intervention studies, including gray literature such as MSc and PhD theses, to overcome publication selection bias. We looked for psycho-socio-spiritual interventions for children with cancer in resource-rich developing countries to compare the impact of resource availability; however, we could not locate peer-reviewed or gray literatures that report effect sizes in such settings.

#### 2.1.3 Data screening and extraction

All search hits including a manually added article were added to Endnote v9.3 where duplicates were deleted, and screening of titles and abstracts was carried out independently by two authors (HM and TNA). Non-English studies were reviewed by a member of the team who speaks the native language of the article. Full-text screening was then carried out by all authors, and data extraction was carried out according to the coding protocol. The reasons for exclusion were recorded, and any disagreements were resolved collectively.

#### 2.1.4 Coding procedure

Data extraction and coding were performed by two authors (TNA and OB). All studies were coded for outcome measures, design and study features, psycho-socio-spiritual intervention, participants’ characteristics, and assessment tools. Disagreements were discussed among the whole team, and unless stated otherwise, a consensus was agreed among all authors.

#### 2.1.5 Outcome measures

The primary outcomes were related to the impact of psycho-socio-spiritual intervention on the outcome of cancer management. Secondary outcomes of interest, however, were related to the role of resources and reforms in psycho-socio-spiritual care provision. In each study, all outcomes related to the impact of psycho-socio-spiritual interventions on physical, psychological, or social wellbeing were captured at different assessment times. The effect size of all interventions and control conditions within each study were coded to enable the calculation of effect sizes, and the instruments used for these measurements were also documented.

#### 2.1.6 Design and study features

The following have been coded: year of publication, type of control group, sample size, country classification, study design, and risk of bias.

##### 2.1.6.1 Psycho-socio-spiritual interventions

The psycho-socio-spiritual interventions across studies exhibited variation in their type, structure, and duration and were coded based on intervention type, delivery setting, provider involved, session length, frequency, and underlying theoretical goals.

##### 2.1.6.2 Participants’ characteristics

We coded for the age of participants, gender, disease type, individual targeted, number of participants, and number of participants as the control group.

##### 2.1.6.3 Assessment tools

As we only included self-reporting tools, we coded the tools utilized across the studies, such as scales, inventories, questionnaire, and indices. A total of 11 different types of tools were used: 34% assessed anxiety, 13% assessed fatigue, 8% assessed QoL, pain, and treatment abandonment, 5% assessed personal adjustment, sleep, burnout, hope, and depression, and 3% assessed the level of joy.

##### 2.1.6.4 Risk of bias assessment and small studies effect

Risk of Bias v2.00 (RoB2) was employed by two authors (HM and HDM) to assess the risk of bias of the included studies. Disagreements were resolved by discussion among the whole team. The following domains were assessed: selection bias, performance bias, detection bias, attrition bias, reporting bias, and other bias. The risk in each domain and the overall risk for the study were judged as low, moderate, or high. The bias due to small studies’ impact was assessed via the funnel plot visual and quantitative analysis.

##### 2.1.6.5 Meta-analysis quality

We adhered to AMSTAR 2 and PRISMA guidelines to ensure a high-quality reporting of this meta-analysis (38, 39). At least two authors assessed the quality according to the tools’ guidelines, and any disagreement was discussed among the whole team.

##### 2.1.6.6 Study heterogeneity and variance components

In order to find sources of heterogeneity, the list of continuous and categorical moderators was coded for, as listed in **Table 1**. Heterogeneity was assessed using several steps, and contrary to other studies where heterogeneity is wrongly quantified using the *I* ^2^, the *Q*, or the *p* statistics (40), we employed the prediction interval equation (**Supplementary Material Appendix B**) to identify the degree of dispersion of true effect of psycho-socio-spiritual interventions and a meta-regression analysis based on the method of moments estimator to determine where interventions are harmful or beneficial and to what degree (41, 42). We established a meta-regression model to explore the sources of heterogeneity and predict the future outcomes as follows:

Testing each of the 20 covariates listed in **Table 1** individually to check the proportion of variance that it explains.

Testing all the covariates that explained a significant proportion of the variance simultaneously.

The *Q*, *df*, and *p* statistics were used to test the null hypothesis that states: “There is no variation at all in the effect size between studies, and any observed variation is purely due to sampling error. If the *Q*-value is larger than the *df*, then the variance between studies will be positive and estimated to be bigger than zero. The popular *I* ^2^ percentage statistic was correctly used to determine the percentage of true effect of the interventions and determine the inflation of observed effect due to sampling error. Importantly, *I* ^2^ was not used to categorize heterogeneity into small, moderate, or large (40, 43–49).

The model’s goodness of fit analysis, the *Q*, the *df*, and the *p* statistics were employed to test the proportion of unexplained variance and accept or reject the null hypothesis that the unexplained variance is zero. A *p*-value of >0.05 confirms the null hypothesis, and the residual variance in true effect will be equal or very close to zero.

The total and residual variance in true effect after applying the model was tested, and the residual *T* ^2^ was calculated to determine the proportion that is not explained by the model. The *R* ^2^ statistic determined the percentage of the variance that was left unexplained after applying the model according to the following equation *R* ^2^ = (total variance - residual variance)/(total variance). A value of 1 = 100% of the true variance was explained by the model.

##### 2.1.6.7 Creating a simulation of the model

The full factorial fit model design analysis of SAS JMP software v. 17.3 was employed to design a model of all included covariates, and the simulation was saved as an interactive html for use by investigators (**Supplementary Material Appendix C**).

##### 2.1.6.8 Data synthesis and analysis

Pooled effect sizes, ANOVAs, moderators, heterogeneity, and publication bias analyses were performed via the Comprehensive Meta-Analysis software (CMA-v2), while the quality of included studies was assessed via Cochrane’s RoB2, and the simulation of the meta-regression model was created by the SAS JMP v17.3 software.

Primary outcomes were assessed through a weighted random effect model. Anxiety and depression, fatigue, sleep quality, distress, or burnout, quality of life, treatment completion or abandonment, and pain reduction were compared between control and intervention groups. The log OR was employed as effect size to ensure normalizing the distribution of the included sample, and wherever needed, the corresponding OR value was mentioned to ease the interpretation of the effect size.

Sub-studies were grouped and compared together using a mixed-model analysis; a random model was employed to identify the group effect size using a common variance (*T* ^2^) and ensure generalizability and a fixed-effect model to avoid wrongly considering grouping as a randomization process. The *p*-statistic was utilized to accept or reject the null hypothesis (41).

## 3 Results

**Figure 1:**
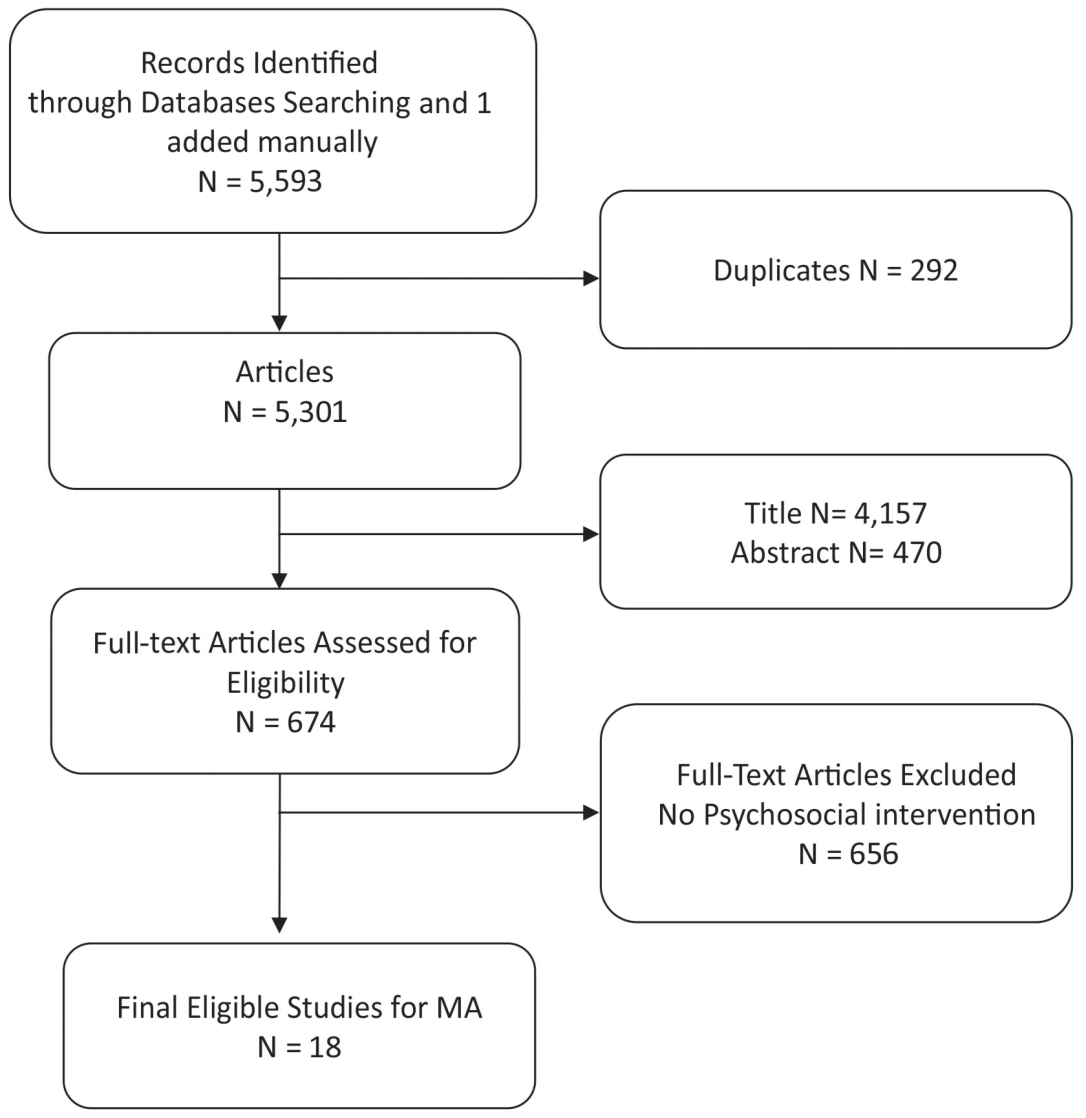
PRISMA flow chart showing the flow of searching, screening, and selecting the eligible studies for this meta-analysis. Out of the 5,953 hits, 18 studies were eligible for inclusion in the meta-analysis.

Screening of 5,301 hits was conducted by two independent researchers (TNA and OB) after excluding 292 duplicates of the total of 5,593 hits (5,398 full articles and 195 MSc/PhD theses). Furthermore, 674 full texts were screened after excluding 4,157 titles and 470 abstracts. A total of 18 eligible studies (50–66) (**Figure 1**) were included in the quantitative meta-analysis and covered a sample size of 3,072 (1,372 children with cancer and 1,700 family members) in seven countries (**Table 2**).

**Table 2:** 18 studies investigated the impact of a wide range of psychosocial interventions on various outcomes (anxiety, depression, fatigue, distress, burnout, quality of life, social functioning, treatment completion and pain reduction).

S/N	Study identifier	Study design	N	Targeted/Age	Child Ages	Intervention	Therapeutic goal	Measured outcome	Assessment tools	Funding	LMIC	Type of cancer
1	Abdullah 18	Randomized Controlled Trial	60	Patients	7 - 16	Creative art therapy	Improve QoL via art therapy	Depressive mood, emotions, and stressful feelings Overall health, Physical activity and Enjoying leisure time and participate in social activities	Kidscreen 10 Index	Not mentioned, No conflict of interest	Iraq	Mixed cancers
2	Ahmadi 18 ^(L)^	Clinical Trial	99	Mothers/26-38	2 - 10	Writing 20 minutes prayers	Reduce anxiety via writing prayers	Anxiety Score	Trait/State Anxiety Inv	Not mentioned, No conflict of interest	Iran	Mixed cancers
3	Alam 19	Retrospective	1042	Family	< 18	Financial +/- counselling	Reduce abandonment via financial support and group counselling	Abandonment of treatment	Rate calculation	Not mentioned, No conflict of interest	India	Acute Lymphocytic Leukemia
4	Alavi 21 ^(L)^	Quasi-experiment Longitudinal	30	Patients	8 - 12	Cognitive play therapy	Reduce pain and improve hope via play therapy	Hope (Snyder’s Hope Scale), passive, active and adaptive pain response and & adjustment (Dokhanchi Children Adjustment)		Khoamshahr-Persian Gulf Islamic Azad University	Iran	Acute Lymphocytic Leukemia
5	Alparlslan 12	Quasi-experiment descriptive	90	Mothers and siblings	9 - 18	Nursing support	Reduce anxiety via nursing support to develop coping mechanisms	Anxiety Score	Trait/State Anxiety Inv	Not mentioned, No conflict of interest	Turkey	Mixed cancers/chronic hematology
6	Altay 17	Quasi-experiment	30	Patients	9-16	Drawing, writing and story telling	Reduce anxiety via drawing, writing, storytelling	Anxiety Score	State Anxiety Inv	Not mentioned, No conflict of interest	Turkey	Mixed cancers
7	Alvarez 17	Retrospective	998	Patients	0 - 18	Psychosocial team intervention	Reduce abandonment via multidisciplinary psychosocial intervention	Abandonment of treatment	Rate calculation	Donations through “Help me to Live Foundation”	Guat-emala	Mixed cancers
8	Bahrami 19 ^(L)^	Randomized Controlled Trial	60	Parents	3 - 12	Emotion regulation	Reduce anxiety via emotional regulation training	Anxiety Score	Beck Anxiety Inv	Not mentioned, No conflict of interest	Iran	Mixed cancers
9	Behesht-ipour 16 ^(L)^	Randomized Controlled Trial	135	Parents	6 - 12	Educational spiritual intervention	Reduce burnout via educational program	Parents Burnout (Shirom and Melamed Burnout Questionnaire)		Not mentioned, No conflict of interest	Iran	Mixed cancers
10	Li 23	Randomized Controlled Trial	99	Patients	8 - 14	Child Life Services (CLS)	Reduce anxiety, pain and fatigue and improve sleep quality via CLS	Pain, Anxiety, Fatigue and Sleep hygiene (Pediatric patient reported outcome measures)		Grant No. 72104167 Youth Science Found. and Grant No. SKY2021048 Suzhou Science and Technology Plan Project	China	Acute Lymphocytic Leukemia
11	Nazari 14	Quasi-experiment	10	Mothers	0 - 15	Psychoth. spiritual, beliefs and emotions	Reduce anxiety via supportive psychotherapy	Anxiety reduction and openness in discussing problems	Kettles’ anxiety Q	Not mentioned, No conflict of interest	Iran	Acute Lymphocytic Leukemia
12	Nicksere-sht 16 ^(L)^	Quasi-experiment	25	Mothers/20 - 50	6 - 18	Education (medical and spiritual) interv.	Reduce anxiety and depression via spiritual counseling and support	Anxiety, depression, and overall mental health	General Health Q.	Not mentioned, No conflict of interest	Iran	Mixed cancers
13	Pouraboli 19	Randomized Controlled Trial	120	Parents/ 27 - 47	N/A	Benson Relaxation technique	Reduce anxiety and fatigue and improve sleep quality via relaxation	Anxiety (Trait/State Anxiety Inv), Fatigue (Brief fatigue inventory) and Sleep quality (Pittsburgh sleep Inventory)		Not mentioned, No conflict of interest	Iran	Acute Lymphocytic Leukemia
14	Pourmo-vahed 13	Randomized Controlled Trial	100	Patients	6-15	Breathing regulation	Reduce procedural pain via breathing regulation	Pain score	Wong pain face scale	Not mentioned, No conflict of interest	Iran	Acute Lymphocytic Leukemia
15	Safarab-adi 16	Randomized Controlled Trial	62	Parents/ 24 - 47	2 - 12	Education, stress and coping management	Improve QoL via a Brief Psychosocial intervention	Caregiver Quality of Life Index, Mental/Emotional burden, lifestyle disruption and Total Quality of Life score		Not mentioned, No conflict of interest	Iran	Mixed cancers
16	Shekraa-bi 12	Semi-experimental research	20	Mothers	2 - 12	Hope therapy	Reduce depression and improve hope via hope group therapy	Hope (Snyder’s Hope Scale) and Depression (Beck depression scale)		Not mentioned, No conflict of interest	Iran	Mixed cancers
17	Sriasih 19	Quasi-experiment descriptive	58	Patients	6 - 12	Music and education (Sleep Hygiene)	Reduce fatigue and improve sleep quality and functionality via music and sleep hygiene therapy	Fatigue (Allen’s fatigue in children cancer scale), Sleep (Sleep problems in children scale and functionality (Barthel status)		DRPM Universitas Indonesia No. 1832 UN2.R3.1/HKP.05.00/2018	Indonesia	Mixed cancers
	Zhang 19	Randomized Controlled Trial	37	Parents	0 - 13	Solution focused brief therapy	Improve resilience and mood via cognitive behavioral therapy	Anxiety and depression (Brief symptoms inventory) and Parental distress (Brief symptoms inventory)		Chongqing Health and Family Planning Commission no. 2017ZDXM013	China	Mixed cancers

Only seven countries (10 studies from Iran, 2 from Turkey, 2 from China and 1 each from, Guatemala, India, Indonesia, and Iraq) showed evidence of psychosocial interventions. Children’s ages ranged from 0-18 years and parents’ age ranges were 20-50 years. Two retrospective cohort or chart review studies (3 and7), Six single-group pre- and post- design experimental studies (4, 5, 6, 11, 12, and 17), and nine randomized clinical trials (1, 2, 8, 9, 13, 14, 15, and 18) with a quality of evidence rated as 2B. The studies employed 17 different assessment tools to report the above-mentioned outcomes. Six studies recruited children with cancer (n = 1276), nine recruited parents only (n = 1610, 4 of which comprised mothers only) and one recruited mothers and siblings (n = 90).

Samples were homogenous in studies 4, 11a nd 14 as recruited children with acute lymphocytic leukemia, or their mothers.

L, longitudinal design. Psychoth, psychotherapy. Interv, intervention.

### 3.1 Impact of psycho-socio-spiritual interventions on the outcomes for children with cancer

**Figure 2:**
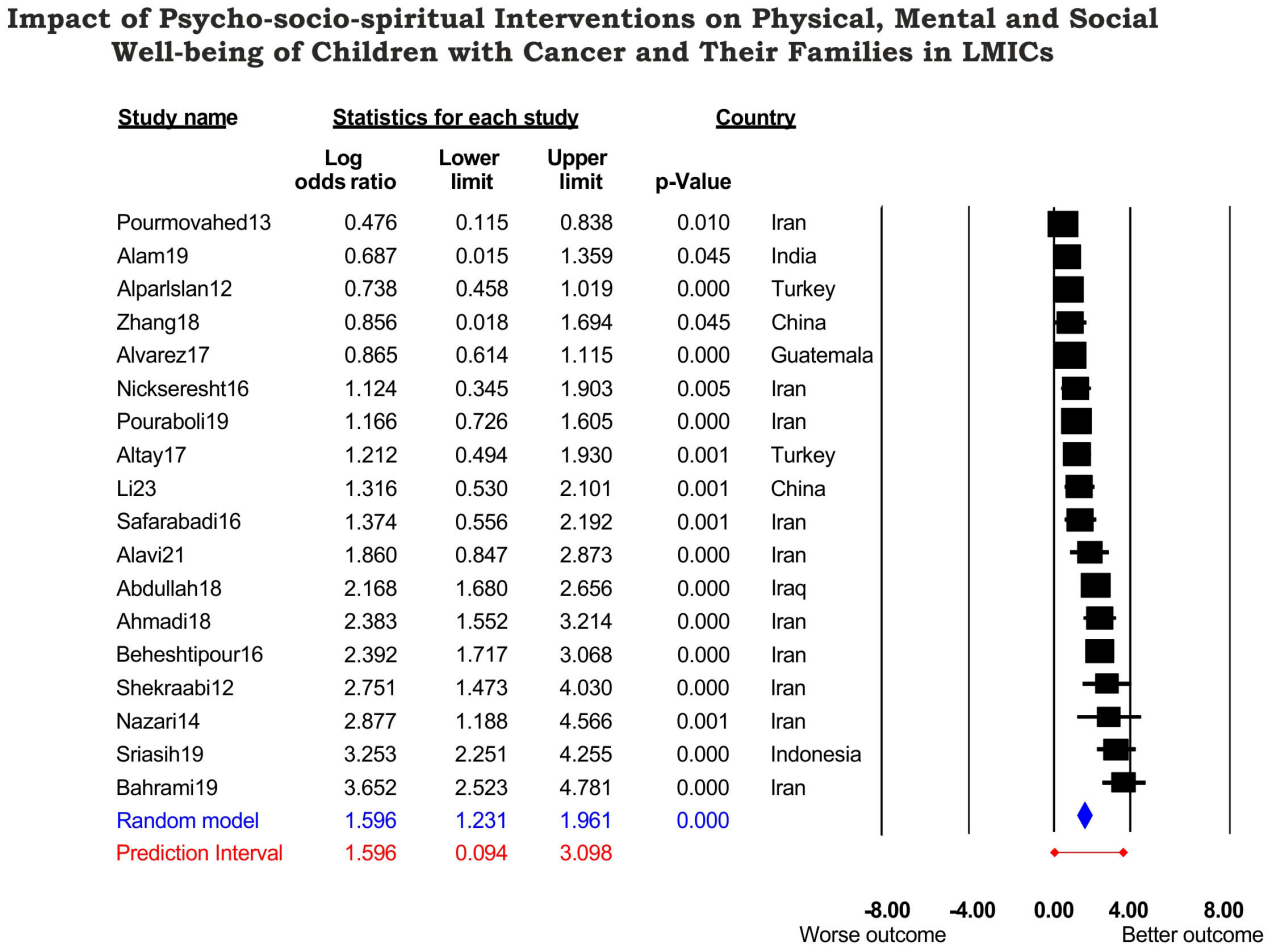
Forest plot showing the impact of the 18 studies on the wellbeing of children with cancer and their families in seven countries that are classified as LMICs by the World Development Bank. A significant observed effect size of log OR 1.596 corresponds to an OR of 4.93. The true effect size in log OR was predicted between 0.094 and 3.098 (OR 1.231–22.148) and indicated a wide dispersion of true effect; some patients did not benefit, some had moderate to extreme benefit from interventions. However, the PREDICTION INTERVAL (*C*) did not cross the zero mark, indicating that psychosocial interventions did not harm the patients or family members.

The overall log OR was 1.596 (95% CI: 1.231–1.961, *p <* 0.0001), implying that interventions are approximately five times more likely to enhance the wellbeing of children and families with cancer, (OR: 4.933, 95% CI: 3.423–7.108, *p <* 0.0001) (**Figure 2**). There was no significant difference between targeting patients or family members, *p* = 0.674 (**Table 3**).

**Table 3:** Subgroup analyses strategy utilizing a mixed effect model analysis; a random effect model assuming common variance (*T^2^*) across subgroups, and a fixed effect model analysis between groups to avoid a mistake of considering grouping as a randomization process and the p value was utilized to accept or reject the null hypothesis.

Group	Studies No.	Log *OR*	Lower limit	Upper limit		Corresp *OR*	Variance	Within group *P*	Tau (*T*)	Tau square (*T^2^*)	*Q* value	*df*	Between groups *P*
1. Targeting patients vs family members													
Family	11	1.681	1.165	2.197		5.370	0.069	<0.001	0.683	0.467	0.177	1	0.674
Patients	7	1.511	0.912	2.110		4.532	0.093	<0.001					
2. Offering passive support vs active participation													
Art	3	1.778	1.150	2.406		5.918	0.103	<0.001	0.730	0.533	0.875	1	0.350
Support	4	1.319	0.590	2.048		3.740	0.138	<0.001					
3. Including the supportive care offered to family in the analysis													
Art	3	1.778	1.150	2.406		5.918	0.103	<0.001	0.683	0.467	0.340	1	0.560
Support	15	1.557	1.163	1.952		4.747	0.040	<0.001					
4. Measuring physical vs psychological outcomes													
Both	7	1.784	1.117	2.451		5.497	0.067	<0.001	0.683	0.467	1.733	2	0.420
Physic	5	1.265	0.541	1.989		3.422	0.105	<0.001					
Psych	6	1.782	1.097	2.467		6.276	0.195	<0.001					
5. Comparing longitudinally vs cross sectional studies													
Long	6	2.094	1.337	2.850		8.116	0.149	<0.001	0.698	0.487	3.031	1	0.082#
Cross	12	1.340	0.955	1.725		3.890	0.039	<0.001					
6. Comparison at follow up													
T1	5*	2.263	1.493		3.034	9.613	0.155	<0.001	0.690	0.476	4.516	1	0.034
T0	13	1.339	0.974		1.736	3.814	0.035	<0.001					

1.) There were 7 studies that examined the impact on patients and 11 studied the impact on family members showed a *p* value of 0.674 indicating an accepted null hypothesis indicating no difference in effect size between the patients and family members. Patients who receive psychosocial interventions (n=7) are 4.5 times likely to have a better wellbeing than those who don’t. Their family members (n=11) are 5 times more likely to have a better wellbeing as well.

2.) Two types of interventions could be compared, play/art therapy (drawing, writing or story telling) and supportive care (nursing, spiritual, financial, counselling, education, relaxation or emotional). Patients who receive art therapy (n=3) or supportive therapy (n=4) are 5.9 or 3.7 times likely to have a better wellbeing respectively than those who don’t. However, the difference was not significant p=0.35.

3.) Adding the 11 studies that offered supportive care to the family (n=15) showed a 4.7 folds improvement in wellbeing and did not reverse the insignificance p=0.560.

4.) Interventions that focused on physical well-being (n=5) or psychosocial QoL (n=6) showed improvement in QoL by 6.276 and 3.422 folds respectively, while combining different types of interventions(n=7) to target physical and psychosocial well-being were not significantly superior (OR 5.497, p=0.42).

5.) The longitudinally designed interventions had a stronger impact, children and families are 8 folds likely to have a better well-being at a marginally significant level p=0.082 (# rejecting the null hypothesis could represent a type II false negative error.

6.) The benefit of psychosocial interventions continued to develop over time to show a significant difference at long term follow up. *Alavi studied the physical and psychosocial impact at T0 and T1, hence 5 studies were considered at T1 and 13 at T0.

Corresp, Corresponding; Long, Longitudinal; Cross, Cross sectional; Physic, Physical; Psych, Psychosocial.

### 3.2 Do outcomes differ across types of psycho-socio-spiritual intervention or targeted outcome?

Three studies (1, 4, and 6) examined the impact of art therapy on children, and four studies (7, 10, 14, and 17) examined the impact of supportive care on children, and the comparison showed *p* = 0.350. Adding the 11 other studies (2, 3, 5, 8, 9, 11, 12, 13, 15, 16, and 18) that examined the supportive care for family members did not change the significance pattern (*p* = 0.560), and no difference between art therapy or supportive care was identified (**Table 3**). The intervention was further classified into a categorical moderator of passive support and active participation (**Table 1**).

A classical classification of QoL sorts it into physical and psycho-socio-spiritual domains (6), and five studies focused on the physical outcome (3, 7, 9, 11, and 14), six focused on psycho-socio-spiritual wellbeing (2, 5, 6, 8, 16, and 18), and seven examined both (1, 4, 10, 12, 13, 15, and 17) There were no significant differences between interventions combined or uncombined (*p* = 0.420), and thus the null hypothesis was accepted. This subgroup was further classified into a categorical moderator to report a single vs. multiple outcome (**Table 1**).

### 3.3 Outcomes by study design

Interestingly, longitudinally designed studies (*n* = 6, OR = 8.116, *p <* 0.001) (2, 4, 8–10, and 12) had a greater effect size than cross-sectional studies (*n* = 12, OR = 3.890, *p <* 0.001) (1, 3, 5–7, 11, and 13–18). The difference between the two groups showed *p* = 0.082 at time point 0, which probably reflects a type II error due to the small number of participants in some studies (90% study power requires a minimum of 85 participants per study, which was not the case for studies 1, 6, 11, and 15–18) (**Table 3**).

The benefit continued, indicating that children and families who undergo structured and repeated psycho-socio-spiritual interventions are 9.613 times more likely to experience a longer-lasting impact (**Table 3**). This was further explored in a meta-regression analysis through studying two variables: the dosing of interventions and the quality of study design (**Table 1**).

### 3.4 Diversity of the effect of psycho-socio-spiritual care

The *I* ^2^ percentage value for the 18 studies was 85.48%, which indicated that 85% of the variance in observed effect reflects variance in true effect, and only 15% were due to sampling errors.

The *Q*-value was 117.11, *df* = 17, and *p* < 0.001, and thus the null hypothesis was rejected, and the true effect size varied between studies.

Assuming a normally distributed population, the prediction interval in log OR (*C* = 1.596 in 95% of all comparable populations fall in the interval of 0.094–3.098), 10% of the sample experienced negligible to low impact, 75% of the population experienced moderate–high benefits, and 10% experienced a very high impact (**Supplementary Figure S1**). Nevertheless, the prediction interval value did not cross the zero mark, and psycho-socio-spiritual interventions thus were unlikely to harm children or families with cancer.

### 3.5 Explanatory model of heterogeneity and its simulation

The model that explained the between-study variance comprised 10 covariates: three continuous and seven categorical covariates (**Table 1**, **Supplementary Figure S6**).

The overall simultaneous analysis of all covariates yielded a *Q*-value of 110.49, *df* =12, *p* = 0.000001, concluding that the model explained some of the variance in true effect. The value of *I* ^2^ *was* 85.25%, indicating that 85% of the between-study variation was due to true effect and that only 15% was due to sampling errors. The prediction interval conversely showed a wide dispersion of true effect so that the impact of intervention spanned a spectrum of no benefit to a very-high-impact one.

The goodness of fit analysis of the model showed no residual of variance about the regression line *T* ^2^ = 0.00, a standard deviation about the regression line *T* = 0.00, and *Q* = 4.74, *df* = 4, and *p* = 0.449, which confirmed the null hypothesis that the unexplained variance in true effect between studies was zero.

The variance of true effects about the grand mean was 0.4671, and the residual variance about the regression line was 0.00. Thus, this proposed model could explain all the between-studies variances of true effects, *R* ^2^ = 1 (100%), *p <* 0.000000001.

It is widely variable, but some studies reported a small yet significant impact—log OR 0.476, 95% CI: 0.115–0.838, *p* = 0.01 (OR 1.610) (study 14), while others reported higher observed effects—log OR 0.687 and 0.856 (OR 1.987 and 2.353). The lower limit of 95% CI was 0.015 and 0.018, respectively (studies 3 and 18). However, the majority (1, 2, 4–13, and 15–17) showed a moderate to high impact, with log OR ranging from 0.738–2.877 (OR 2.092–17.762). Furthermore, evidence from Indonesia and Iran reported a very high impact—log OR 3.253 and 3.652 (OR 25.867 and 38.557), respectively (studies 8 and 17) (**Figure 2**).

The proposed simulation model encompassed 491,520 possible combinations to explain the heterogeneity, predict outcomes of future studies, and rank the covariates according to significance. In this order of significance, each unit increased in quality design, the effect size increased by 0.1769, and each unit increased in number of sessions, increasing the effect size by 0.0716 while controlling for the first covariate. A single outcome reporting increased the effect size by 1.2885, with a quasi-experimental design increasing it by 2.4348 while controlling for previous covariates. For cancers requiring shorter treatment durations, a focus on encouraging active participation increased the effect size by 0.8907, and each unit decreased in duration%, increasing the effect size by 0.0498 while controlling for preceding covariates. Studies free of bias increased the effect size by 2.4542, targeting family members increased the effect size by 0.4006, and the absence of medical reform increased the effect size by 0.6114 while controlling for all covariates (**Supplementary Figure S2**, **Supplementary Material Appendix C**).

Retrospective studies, with a concern of bias, requiring longer periods of treatment, and passively supported with long hours, showed no benefit from the intervention. In the absence of risk of bias, randomized studies of children with cancers requiring shorter periods of treatment and encouraged to actively participate through focused sessions with shorter duration% and which reported a single outcome were likely to show a very high impact from the intervention.

### 3.6 Quality of this meta-analysis and risk of bias

Visualization of the funnel plot suggested the presence of bias as the studies were more clustered toward the right side of the mean. The presence of bias was confirmed via Eggers regression intercept (3.617, 95% CI: 1.669–5.565, *df* = 16, *p* = 0.0018) and Begg and Mazumdar correlation of adjusted *T* from 0.451 to 0.444, *z* value from 2.613 to 2.575, and *p* for adjusted *T* of 0.01.

However, Duval and Tweedie’s trim-and-fill analysis under the random-effects model added three presumably missing studies to the left of the mean and confirmed the insignificant change of the log OR (1.36346, 95% CI: 0.99511–1.73181, *p* = 0.371) (**Supplementary Figure S3**).

In contrast, the insignificance of publication bias was objectively confirmed via the fail-safe N and (5 × 18) +10 benchmarking. Rosenthal’s missing studies needed to bring *p* to >0.05 was 1,713, which markedly exceeded the benchmarking value of 100, indicating that this meta-analysis results are robust against the emergence of negative studies in the future (67).

One study remove analysis ruled out existence of outliers that could disproportionately influence the overall result (**Supplementary Figure S4**).

The risk of bias assessment via the RoB2 tool of the Cochrane showed the existence of concern in a few studies that are included in this meta-analysis, and this existence of bias was employed in the meta-regression analysis (**Supplementary Figures S5**, **S6**, **Table 1**).

## 4 Discussion

A significant gap in the field of psycho-socio-spiritual interventions for children and families with cancer in LMICs was covered. This pivotal meta-analysis highlighted the impact of psycho-socio-spiritual care on the wellbeing of children and families with cancer in resource-constrained settings.

Most importantly, it confirmed the poorer physical, mental, and social outcomes of absence of psycho-socio-spiritual support, a concerning finding given that 80% of children who are diagnosed with cancer globally reside in LMICs. Addressing this gap is crucial, as psycho-socio-spiritual support can reduce treatment abandonment, improve adherence, and ultimately enhance the survival and quality of life for the majority of children with cancer in LMIC settings.

Alarmingly, only 10% of LMICs address the psycho-socio-spiritual wellbeing of children with cancer despite a striking improvement in outcome by more than fourfold should psycho-socio-spiritual support be provided. This suggests that children with cancer in 90% of LMICs encounter a twofold disadvantage of lower medical care and absence of psycho-socio-spiritual support, and thus the outcome of their cancer management is roughly five times worse than that of their counterparts. This finding alone is sufficient to require the mandatory provision of holistic psycho-socio-spiritual care to improve the treatment and wellbeing outcomes for a large proportion of children globally.

There is a need to address the lack of resources that can prioritize and balance between treating physically observable symptoms and holistically addressing unmet needs in a context- and culture-sensitive approach that deals with the stigma attached to psychological support.

We believe that this paucity in LMICs is not only due to limited resources, as evidence of existing psycho-socio-spiritual care for children with cancer in countries that are resource-rich was also lacking, suggesting that this deficit is due to a combination of variables.

Moreover, the threefold reported mortality due to the abandonment of treatment could be attributed to poor engaging skills rather than passive financial support, and in such cases, advances of medical management would be of limited value if not accompanied by specialized actively engaging psycho-socio-spiritual programs.

Studies from Iran consistently reported an extremely high OR (up to 96-fold) at a highly significant level. Iran introduced mental health services reform in the early 2000s as well as psycho-socio-spiritual support for cancer patients. Although their reports suggest that this reform improved the access to quality services, reduced the stigma, and enriched the mental services (68–70), our model suggests a negative correlation between reforms and wellbeing. This incidental finding is puzzling in two ways: It illogically suggests that reform harms patients and this covariate was the one to boost the residual explained variance from 81% to 100%. Healthcare reforms, thus, cannot and should not be ignored, and our meta-analysis pointed that out.

Indeed partial, ineffective, non-holistic, contextually incongruent reforms could be more catastrophic than beneficial (8, 34, 71–73). This finding will direct our future research and promotion of psycho-socio-spiritual care for the pediatric population in general and for children with cancer in particular.

Importantly, the possibility of these studies to be outliers or challenged in the future have been ruled out by one study that has been removed, Fail-safe N and the 5K+10 analyses. Moreover, the existence of heterogeneity within studies included in this meta-analysis reflected a true effect and diversity in psycho-socio-spiritual interventions.

More importantly, we avoided a very common oversight of measuring heterogeneity wrongly through *I* ^2^, *Q*, *df*, *p*, or interim variance results. We were among the very few studies that assessed heterogeneity correctly instead through the prediction interval to capture the degree of dispersion of true effect.

This wide dispersion of true effect was analyzed through a multivariate meta-regression and allowed the offering of a simulation model of 10 covariates to test the heterogeneity and predict the outcomes for future studies through >400,000 possible combinations to improve the provision of psycho-socio-spiritual care for children with cancer in LMIC settings.

An important aspect of heterogeneity is the impact it casts on the true effect of the meta-analysis. Our true effect analysis enabled us to identify which environments would cast no impact of high impact on the wellbeing of children with cancer and their families in LMICs’ settings. Retrospective studies, with a concern of bias, requiring longer periods of treatment, and passively supported with long hours, showed no benefit from the intervention. Absence of risk of bias, randomized studies of children with cancer requiring shorter periods of treatment, encouraged to actively participate through focused sessions with shorter duration%, and who reported a single outcome were likely to show a very high impact from the intervention. However, this true effect analysis does not limit the impact of heterogeneity on this meta-analysis (see the discussion in “Limitations”).

As PRISMA and AMSTAR-2 guidelines were followed to ensure high quality, we registered the study, published a protocol, summarized the results in full transparency and provided robust summaries of the intervention’s impact in a large sample of children with cancer by utilizing a random effect model to ensure generalizability across wider contexts.

Moreover, the publication bias was visualized and objectively quantified, and its insignificant impact was confirmed.

Although we needed to include observational non-RCTs due to the scarcity of related interventions in LMICs, the Cochrane RoB2 rated most of the included studies as 2B and allowed employing the randomization and risk of bias as moderators for designing future studies.

## 5 Limitations

There is a limited number of LMICs that implement psychosocial intervention for children with cancer. The psycho-socio-spiritual services represent a wide range of interventions, and the limited number of studies in LMICs forced us to combine a set of heterogenous studies and did not allow for a detailed analysis of moderators and subgroups. It must be noted that LMICs are not comparable with other well-established resourceful countries where hundreds of studies could be reviewed. In this meta-analysis, the limited number of studies did not allow a meaningful subgroup analysis, and we had to resort to a predictive simulation analysis to compensate for this limitation.

The heterogeneity in this meta-analysis, however, came from different sources: clinical, methodological, and statistical aspects.

Clinically, the participants varied widely (age, developmental stage, disease type, and geographical and socio-economical contexts). The type of interventions (passive vs. targeting functionality), their intensity, frequency, and providers also varied widely, and a meaningful subgroup analysis could not be carried out due to the low number of sub-studies (a minimum of 10 studies are needed). Although the assessment of outcome varied with regard to the domain of QoL and the assessment tool, we managed to include the dosing of intervention as a significant moderator that was employed in the simulation model to design impactful interventions and research studies.

Methodologically, heterogeneity originated from differences in study design (longitudinal, cross-sectional, randomized, and quasi), which could affect the causal relationship. The sample size also contributed, as smaller studies lacked sufficient statistical power to detect significant effects, increasing the susceptibility to type II errors.

Statistically, heterogeneity was evident due to variability in true effect size beyond chance. The *I* ^2^ value indicated that 85% of the variability originated from true differences across studies.

However, to address this limitation of significant heterogeneity and contrary to common but misleading statistics, we employed several approaches. The prediction interval assessed the dispersion of true effects to reveal the spectrum of negligible to very high impact. The predictive modeling simulation and the multifactorial meta-regression compensated for the small number of studies in LMICs and identified 10 cofactors that explained 100% of the true variability between studies.

The existence of bias due to including studies with insignificant small effect sizes was confirmed visually through inspecting the funnel plot and statistically Eggers regression. However, the robustness of the results of this meta-analysis was confirmed through multiple testing, i.e., the trim-and-fill, the one-study-removed, the classic fail-safe-N, and Rosenthal’s benchmarking analyses. Those besides the above-mentioned simulation and multifactorial meta-regression strengthened the reliability and applicability of the findings across diverse LMICs settings despite the low number of published studies in such contexts.

Additionally, we could not locate published quantitative data from resource-rich developing nations despite our knowledge of the existence of healthcare strategies and future visions in these countries (35, 36). These projected strategies could not be used in our meta-analysis due to the lack of quantitative data and the qualitative studies that raised the number of LMICs with psycho-socio-spiritual services to 11 countries (35, 36, 74–77).

As we excluded the provider perspective reporting from this meta-analysis to focus on the self-reporting patient experience outcome, we could have added to the bias in selection identified in this meta-analysis, and we thus recommend that future studies include provider perspectives in the analysis and use it as a moderator rather than excluding them.

Psycho-socio-spiritual services such as art and music therapy, procedural preparation and education, support for developmentally appropriate, and short- and long-term coping mechanisms for patients and families are readily available in resource-rich countries (78) and are provided to minimize the negative developmental impact of healthcare for children with cancer (79). However, LMICs may not have access to diverse resources for the proactive provision of psycho-socio-spiritual care services including materials and personnel (80, 81). As such, healthcare systems may need to utilize resources based on what is readily available as opposed to what they identify as valuable to patients, and the simulation model that we propose can serve as a tool to identify the best utilization of available resources. Furthermore, children of differing chronological and developmental levels require psycho-socio-spiritual interventions suited to their cognitive abilities in order to promote optimal development. Therefore, psycho-socio-spiritual interventions are framed as a goal directed rather than conducted from a specific psychological theoretical orientation in order to facilitate necessary adaptation to the unique developmental presentation of each patient being served.

It is worth noting that out of the 18 studies, 10 studies originated from Iran and another four from other Islamic countries, which highlights a clustering of geographical and cultural concentration that could have been explored as a categorical moderator in the meta-regression. However, the scarcity of studies from non-Islamic countries in this meta-analysis limited the feasibility of such categorization. Moreover, it underscores our previous recommendation about the need for geographical diverse contextual trials to identify the impact of spiritual interventions (24). In order to address this gap, we have indeed secured a funding to conduct a randomized controlled trial in our unit to compare the efficacy of psycho-social vs. psycho-socio-spiritual interventions for patients with cancer.

Although we planned to code intervention theoretical underpinnings, we substituted this with a more relevant therapeutic goal coding.

## 6 Key messages and recommendations for future directions

The identified gap presents an opportunity for outreach teams to target under-served populations to fulfill the strategic equity among children with cancer globally and elevate their chances of living healthy lives.

The finding of negative correlation of the healthcare reform despite its significance in the explanatory model necessitates a deeper and thorough look at the needed cost-effective, efficient, contextually relevant, and culturally sensitive reforms. An evidence and gap map are needed to discover the status of interventions in resource-rich developing countries. Additionally, healthcare reforms currently in progress within countries that impact the provision of psycho-socio-spiritual care need to be explored further.

These findings will inform the implementation of our model that theorizes the pediatric patient hierarchy of developmental needs (PPHDN) to optimize quality and quantity in hospital holistic care.

The attached simulation model offers hundreds of thousands of choices to guide the provision of psycho-socio-spiritual interventions for children with cancer in LMICs. These choices can be tailored to specific unique contexts of LMICs on the World Bank list. Moreover, the simulation could be employed to explore the heterogeneity in this study to identify further environments that we might have overlooked.

## Data Availability

The original contributions presented in the study are included in the article/**Supplementary Material**, further inquiries can be directed to the corresponding author/s.

## Supplementary material

Supplementary Figure 1 Comparison between the observed (blue curve) and true effect (red curve) sizes assuming a normal distribution of the population. The prediction interval that reflects the heterogeneity of the true effect showed a wide dispersion of effect size.

Supplementary Figure 2 A still image of the simulation model showing the different parameters that can be altered in the simulation with the 95% CI for the three continuous variables. At least 491,000 different combinations of these covariates could be extrapolated to predict the impact of different combinations.

Supplementary Figure 3 Funnel plot showing visually the existence of a selection bias and the potential absence of three studies under the random effect analysis model. The existence of bias was confirmed by Eggers regression intercept (3.617, 95% CI: 1.669–5.565, *df* = 16, *p* = 0.0018) and Begg and Mazumdar rank correlation of adjusted *T* from 0.451 to 0.444, *z*-value from 2.613 to 2.575, and a *p-*value for adjusted *T* of 0.01. Imputing the three presumably missing studies shifted the point estimate to the left of the mean. However, the trim and fill analysis indicated that the change would not be significant (*p* = 0.3714). The *p*-value was calculated using the *z*-score for the difference between the SE of observed and adjusted point estimates. The *z*-score of 0.8943 corresponded to a two-tailed *p*-value of 0.3714.

Supplementary Figure 4 The one-study-removes analysis showing the impact of removing each study on the overall observed effect size and the relative weight that each study holds. No outlier study is skewing the observed effect size, and all studies are sharing comparatively relative weights of 2%–8%.

Supplementary Figure 5 The risk of bias assessment via the RoB2 tool of Cochrane showing the existence of concern in a few studies that are included in this meta-analysis. This concern is shared in the model that explained the observed heterogeneity between studies (variance in true effect).

Supplementary Figure 6 Meta-regression of true effect vs. the number of sessions, showing that the variance in true effect equals the CI when controlling for all covariates. In the environment of the three studies (3, 14, and 18), patients and families might not benefit from psychosocial interventions. This environment encompasses retrospective studies and those with concerns of bias, involving a low number of sessions and passive support. On the other hand, in the environment of the five studies (2, 8, 11, 16, and 17), psychosocial interventions might have a very high impact. This environment encompasses well-designed studies void of bias, with concise focused sessions that target improving the participation of family members for cancers that require shorter periods of treatment. Under the mixed environment of the remaining studies (1, 4–7, 9, 10, 12, 13, and 15), psychosocial interventions might have a moderate to high impact. This environment encompasses well-designed studies, with shorter hours of intervention, that target the active participation of family members of children with cancer requiring shorter treatment protocols regardless of the number of sessions, reported outcome, reform, RoB, and allocation or number of sessions. However, the simulation model can offer much more combination of choices and show their predicted outcome.
