# Supplementary material for: Alarming findings of psycho-socio-spiritual interventions on physical, mental, and social health for children with cancer and their families in low- and middle-income countries: a meta-analysis
Source: Front Psychiatry. 2025 Apr 28;16:1533599. doi: 10.3389/fpsyt.2025.1533599 (PMC12068859; doi:10.3389/fpsyt.2025.1533599)
Supplement: Supplementary Figure 1 — Comparison between the observed (blue curve) and true effect (red curve) sizes assuming a normal distribution of the population. The prediction interval that reflects the heterogeneity of the true effect showed a wide dispersion of effect size. [file SupplementaryFile1.zip › Appendix B.docx]

Appendix B

The prediction interval (*C*) equation is *C*= *M* ± *t_(dƒ)_*√*V_M_* + *T^2^*,

where *M* is the random effects estimate of the mean,

*t_(dƒ)_* is the critical *t*-value for the degrees of freedom and is calculated using the Microsoft excel function *TINV*(0.05, *dƒ*) where *dƒ*= number of studies-2 (*n-2*),

*V_M_* is the error variance of the mean using random effects weights [*V_M_* = ((upper limit of mean (in Log *OR*) – Mean)/1.96)^2^],

*T^2^* is the estimate of the between-study variance.

The excel worksheet that calculates the prediction interval was downloaded from CMA website and the *C* for *OR* and log *OR* ratios were calculated.
