# Supplementary material for: Alarming findings of psycho-socio-spiritual interventions on physical, mental, and social health for children with cancer and their families in low- and middle-income countries: a meta-analysis
Source: Front Psychiatry. 2025 Apr 28;16:1533599. doi: 10.3389/fpsyt.2025.1533599 (PMC12068859; doi:10.3389/fpsyt.2025.1533599)
Supplement: Supplementary Figure 1 — Comparison between the observed (blue curve) and true effect (red curve) sizes assuming a normal distribution of the population. The prediction interval that reflects the heterogeneity of the true effect showed a wide dispersion of effect size. [file SupplementaryFile1.zip › Appendix A.docx]

Appendix A

Search syntax

## **PUBMED**

((pedia*[title] OR paedia*[title] OR child*[title] OR youth[title] OR young*[title] OR infan*[title] OR adolescen*[title] OR teen*[title] OR toddler[title]) AND (( psycho*[title] OR menta*[title] OR wellb*[title] OR well-b*[title] OR emotion*[title] OR soci*[title] OR counsel*[title] OR child life[title] OR childlife[title] OR child-life[title] OR play*[title] OR therap*[title] OR play specialist*[title] OR health*[title] OR health play specialist*[title] OR art therap*[title] OR music therap*[title] OR sound therap*[title] OR animal therap*[title] OR pet therap*[title] OR complementary therap*[title] OR alternative medicine[title] OR mindful*[title] OR legacy buil*[title] OR rehabilitat*[title] OR cognitive behavio*[title] OR non pharmacolog*[title] OR non-pharmacolog*[title] OR relax*[title] OR patient education[title] OR client education[title] OR support group*[title] OR creativ*[title] OR medical play[title] OR humo*[title] OR laughter therap*[title] OR birthday celebrat*[title] OR treatment complet*[title] OR hospital play[title] OR therapeutic play[title] OR preparation[title] OR hospital familiari*[title] OR patient-centered care[title] OR family-centered care[title] OR patient centered care[title] OR family centered care[title] OR end of life care[title] OR eol[title] OR palliative care[title]) ) AND (( cancer[title] OR oncolog*[title] OR tumo*[title] OR leuk*[title] OR malign*[title] OR neoplasm[title] OR swell*[title] OR lesion*[title])) AND (( emerging countr*[all fields] OR emerging nation*[all fields] OR emerging population*[all fields] OR developing countr*[title] OR developing nation*[title] OR developing population*[title] OR developing world[title] OR less developed countr*[title] OR less developed nation*[title] OR less developed population*[title] OR less developed world[title] OR lesser developed countr*[title] OR lesser developed nation*[title] OR lesser developed population*[title] OR lesser developed world[title] OR under developed countr*[title] OR under-developed countr*[title] OR under developed nation*[title] OR under-developed nation*[title] OR under developed population*[title] OR under-developed population*[title] OR under developed world[title] OR under-developed world[title] OR underdeveloped countr*[title] OR underdeveloped nation*[title] OR underdeveloped population*[title] OR middle income countr*[title] OR middle income nation*[title] OR middle income population*[title] OR middle-income countr*[title] OR middle-income nation*[title] OR middleincome population*[title]OR low income countr*[title] OR low-income countr*[title] OR low income nation*[title] OR low income population*[title] OR low-income countr*[title] OR low-income nation[title] OR low-income population*[title] OR lower income countr*[title] OR lower income nation*[title] OR lower income population*[title] OR lower-income countr*[title] OR lower-income nation*[title] OR lower-income population*[title] OR underserved countr*[title] OR underserved nation*[title] OR underserved population*[title] OR underserved world*[title] OR under served countr*[title] OR under served nation*[title] OR under served population*[title] OR under served world[title] OR deprived countr*[title] OR deprived nation*[title] OR deprived population*[title] OR deprived world[title] OR "poor country"[title] OR "poor countries"[title] OR "poor nation"[title] OR "poor nations"[title] OR "poor population"[title] OR "poor populations"[title] OR "poor world"[title] OR poorer countr*[title] OR poorer nation*[title] OR poorer population*[title] OR poorer world[title] OR "developing economy"[title] OR "developing economies"[title] OR less developed econom*[title] OR lesser developed econom*[title] OR under developed econom*[title] OR underdeveloped econom*[title] OR "middle income economy"[title] OR "middle income economies"[title] OR low income econom*[title] OR lower income econom*[title] OR low gdp[title] OR low gnp[title] OR low gross domestic[title] OR low gross national[title] OR "lower gdp"[title] OR lower gnp[title] OR " lower gross domestic"[title] OR lower gross national[title] OR lmic[title] OR lmics[title] OR "third world"[title] OR lami countr*[title] OR "transitional countr*"[title] OR Africa[title] OR Asia[title] OR Caribbean[title] OR West Indies[title] OR South America[title] OR Latin America[title] OR Central America[title] OR "Atlantic Islands"[title] OR "Commonwealth of Independent States"[title] OR "Pacific Islands"[title] OR "Indian Ocean Islands"[title] OR "Eastern Europe"[title] OR Afghanistan[title] OR Albania[title] OR Algeria[title] OR Angola[title] OR Antigua[title] OR Barbuda[title] OR Argentina[title] OR Armenia[title] OR Armenian[title] OR Aruba[title] OR Azerbaijan[title] OR Bahrain[title] OR Bangladesh[title] OR Barbados[title] OR Benin[title] OR Byelarus[title] OR Byelorussian[title] OR Belarus[title] OR Belorussian[title] OR Belorussia[title] OR Belize[title] OR Bhutan[title] OR Bolivia[title] OR Bosnia[title] OR Herzegovina[title] OR Hercegovina[title] OR Botswana[title] OR Brasil[title] OR Brazil[title] OR Bulgaria[title] OR Burkina Faso[title] OR Burkina Fasso[title] OR Upper Volta[title] OR Burundi[title] OR Urundi[title] OR Cambodia[title] OR Khmer Republic[title] OR Kampuchea[title] OR Cameroon[title] OR Cameroons[title] OR Cameron[title] OR Cape Verde[title] OR Central African Republic[title] OR Chad[title] OR Chile[title] OR China[title] OR Colombia[title] OR Comoros[title] OR Comoro Islands[title] OR Comores[title] OR Mayotte[title] OR Congo[title] OR Zaire[title] OR Costa Rica[title] OR Cote d'Ivoire[title] OR Ivory Coast[title] OR Croatia[title] OR Cuba[title] OR Cyprus[title] OR Czechoslovakia[title] OR " Czech Republic "[title] OR Slovakia[title] OR Slovak Republic[title] OR Djibouti[title] OR French Somaliland[title] OR Dominica[title] OR Dominican Republic[title] OR East Timor[title] OR East Timur[title] OR Timor Leste[title] OR Ecuador[title] OR Egypt[title] OR United Arab Republic[title] OR El Salvador[title] OR Eritrea[title] OR Estonia[title] OR Ethiopia[title] OR Fiji[title] OR Gabon[title] OR Gabonese Republic[title] OR Gambia[title] OR Gaza[title] OR Georgia Republic[title] OR Georgian Republic[title] OR Ghana[title] OR Gold Coast[title] OR Greece[title] OR Grenada[title] OR Guatemala[title] OR Guinea[title] OR Guam[title] OR Guiana[title] OR Guyana[title] OR Haiti[title] OR Honduras[title] OR Hungary[title] OR India[title] OR Maldives[title] OR Indonesia[title] OR Iran[title] OR Iraq[title] OR Jamaica[title] OR Jordan[title] OR Kazakhstan[title] OR Kazakh[title] OR Kenya[title] OR Kiribati[title] OR Korea[title] OR Kosovo[title] OR Kyrgyzstan[title] OR Kirghizia[title] OR Kyrgyz Republic[title] OR Kirghiz[title] OR Kirgizstan[title] OR "Lao PDR"[title] OR Laos[title] OR Latvia[title] OR Lebanon[title] OR Lesotho[title] OR Basutoland[title] OR Liberia[title] OR Libya[title] OR Lithuania[title]OR Macedonia[title] OR Madagascar[title] OR Malagasy Republic[title] OR Malaysia[title] OR Malaya[title] OR Malay[title] OR Sabah[title] OR Sarawak[title] OR Malawi[title] OR Nyasaland[title] OR Mali[title] OR Malta[title] OR Marshall Islands[title] OR Mauritania[title] OR Mauritius[title] OR Agalega Islands[title] OR Melanesia[title] OR Mexico[title] OR Micronesia[title] OR Middle East[title] OR Moldova[title] OR Moldovia[title] OR Moldovian[title] OR Mongolia[title] OR Montenegro[title] OR Morocco[title] OR Ifni[title] OR Mozambique[title] OR Myanmar[title] OR Burma[title] OR Namibia[title] OR Nepal[title] OR Netherlands Antilles[title] OR New Caledonia[title] OR Nicaragua[title] OR Niger[title] OR Nigeria[title] OR Northern Mariana Islands[title] OR Oman[title] OR Muscat[title] OR Pakistan[title] OR Palau[title] OR Palestine[title] OR Panama[title] OR Paraguay[title] OR Peru[title] OR Philippines[title] OR Philipines[title] OR Phillipines[title] OR Phillippines[title] OR Poland[title] OR Portugal[title] OR Puerto Rico[title] OR Romania[title ] OR Rumania[title] OR Roumania[title] OR Russia[title] OR Russian[title] OR Rwanda[title] OR Ruanda[title] OR Saint Kitts[title] OR St Kitts[title] OR Nevis[title] OR Saint Lucia[title] OR St Lucia[title] OR Saint Vincent[title] OR St Vincent[title] OR Grenadines[title] OR Samoa[title] OR Samoan Islands[title] OR Navigator Island[title] OR Navigator Islands[title] OR Sao Tome[title] OR Saudi Arabia[title] OR Senegal[title] OR Serbia[title] OR Montenegro[title] OR Seychelles[title] OR Sierra Leone[title] OR Slovenia[title] OR Sri Lanka[title] OR Ceylon[title] OR Solomon Islands[title] OR Somalia[title] OR Sudan[title] OR Suriname[title] OR Surinam[title] OR Swaziland[title] OR Syria[title] OR Syrian[title] OR Tajikistan[title] OR Tadzhikistan[title] OR Tadjikistan[title] OR Tadzhik[title] OR Tanzania[title] OR Thailand[title] OR Togo[title] OR Togolese Republic[title] OR Tonga[title] OR Trinidad[title] OR Tobago[title] OR Tunisia[title] OR Turkey[title] OR Turkmenistan[title] OR Turkmen[title] OR Tuvalu[title] OR Uganda[title] OR Ukraine[title] OR Uruguay[title] OR USSR[title] OR Soviet Union[title] OR Union of Soviet Socialist Republics[title] OR Uzbekistan[title] OR Uzbek OR Vanuatu[title] OR New Hebrides[title] OR Venezuela[title] OR Vietnam[title] OR Viet Nam[title] OR West Bank[title] OR Yemen[title] OR Yugoslavia[title] OR Zambia[title] OR Zimbabwe[title] OR Rhodesia[title] OR Developing Countries[Mesh] OR Africa[Mesh:NoExp] OR Africa Northern[Mesh:NoExp] OR Africa South of the Sahara[Mesh:NoExp] OR Africa Central[Mesh:NoExp] OR Africa Eastern[Mesh:NoExp] OR Africa Southern[Mesh:NoExp] OR Africa Western[Mesh:NoExp] OR Asia[Mesh:NoExp] OR Asia Central[Mesh:NoExp] OR Asia Southeastern[Mesh:NoExp] OR Asia Western[Mesh:NoExp] OR Caribbean Region[Mesh:NoExp] OR West Indies[Mesh:NoExp] OR South America[Mesh:NoExp] OR Latin America[Mesh:NoExp] OR Central America[Mesh:NoExp] OR "Atlantic Islands"[Mesh:NoExp] OR "Commonwealth of Independent States"[Mesh:NoExp] OR "Pacific Islands"[Mesh:NoExp] OR "Indian Ocean Islands"[Mesh:NoExp] OR "Europe Eastern"[Mesh:NoExp] OR Afghanistan[Mesh] OR Albania[Mesh] OR Algeria[Mesh] OR American Samoa[Mesh] OR Angola[Mesh] OR "Antigua and Barbuda"[Mesh] OR Argentina[Mesh] OR Armenia[Mesh] OR Azerbaijan[Mesh] OR Bahrain[Mesh] OR "Baltic States"[Mesh] OR Bangladesh[Mesh] OR Barbados[Mesh] OR Benin[Mesh] OR "Republic of Belarus"[Mesh] OR Belize[Mesh] OR Bhutan[Mesh] OR Bolivia[Mesh] OR Bosnia-Herzegovina[Mesh] OR Botswana[Mesh] OR Brazil[Mesh] OR Bulgaria[Mesh] OR Burkina Faso[Mesh] OR Burundi[Mesh] OR Cambodia[Mesh] OR Cameroon[Mesh] OR Cape Verde[Mesh] OR Central African Republic[Mesh] OR Chad[Mesh] OR Chile[Mesh] OR China[Mesh] OR Colombia[Mesh] OR Comoros[Mesh] OR Congo[Mesh] OR Costa Rica[Mesh] OR Cote d'Ivoire[Mesh] OR Croatia[Mesh] OR Cuba[Mesh] OR Cyprus[Mesh] OR Czechoslovakia[Mesh] OR Czech Republic[Mesh] OR Slovakia[Mesh] OR Djibouti[Mesh] OR "Democratic Republic of the Congo"[Mesh] OR "Democratic People's Republic of Korea"[Mesh] OR Dominica[Mesh] OR Dominican Republic[Mesh] OR East Timor[Mesh] OR Ecuador[Mesh] OR Egypt[Mesh] OR El Salvador[Mesh] OR Eritrea[Mesh] OR Estonia[Mesh] OR Ethiopia[Mesh] OR " Equatorial Guinea"[Mesh] OR Fiji[Mesh] OR "French Guiana"[Mesh] OR Gabon[Mesh] OR Gambia[Mesh] OR "Georgia (Republic)"[Mesh] OR Ghana[Mesh] OR Greece[Mesh] OR Grenada[Mesh] OR Guatemala[Mesh] OR Guinea[Mesh] OR Guinea-Bissau[Mesh] OR Guam[Mesh] OR Guyana[Mesh] OR Haiti[Mesh] OR Honduras[Mesh] OR Hungary[Mesh] OR "Independent State of Samoa"[Mesh] OR India[Mesh] OR Indonesia[Mesh] OR Iran[Mesh] OR Iraq[Mesh] OR Jamaica[Mesh] OR Jordan[Mesh] OR Kazakhstan[Mesh] OR Kenya[Mesh] OR Korea[Mesh] OR Kyrgyzstan[Mesh] OR Laos[Mesh] OR Latvia[Mesh] OR Lebanon[Mesh] OR Lesotho[Mesh] OR Liberia[Mesh] OR Libya[Mesh] OR Lithuania[Mesh] OR Macedonia* Republic[Mesh] OR Madagascar[Mesh] OR Malawi[Mesh] OR Malaysia[Mesh] OR Mali[Mesh] OR Malta[Mesh] OR Mauritania[Mesh] OR Mauritius[Mesh] OR "Melanesia"[Mesh] OR Mexico[Mesh] OR Micronesia[Mesh] OR Middle East[Mesh:NoExp] OR Moldova[Mesh] OR Mongolia[Mesh] OR Montenegro[Mesh] OR Morocco[Mesh] OR Mozambique[Mesh] OR Myanmar[Mesh] OR Namibia[Mesh] OR Nepal[Mesh] OR Netherlands Antilles[Mesh] OR New Caledonia[Mesh] OR Nicaragua[Mesh] OR Niger[Mesh] OR Nigeria[Mesh] OR Oman[Mesh] OR Pakistan[Mesh] OR Palau[Mesh] OR Panama[Mesh] OR Papua New Guinea[Mesh] OR Paraguay[Mesh] OR Peru[Mesh] OR Philippines[Mesh] OR Poland[Mesh] OR Portugal [Mesh] OR Puerto Rico[Mesh] OR "Republic of Korea"[Mesh] OR Romania[Mesh] OR Russia[Mesh] OR "Russia (Pre-1917)"[Mesh] OR Rwanda[Mesh] OR "Saint Kitts and Nevis"[Mesh] OR Saint Lucia[Mesh] OR "Saint Vincent and the Grenadines"[Mesh] OR Samoa[Mesh] OR Saudi Arabia[Mesh] OR Senegal[Mesh] OR Serbia[Mesh] OR Montenegro[Mesh] OR Seychelles[Mesh] OR Sierra Leone[Mesh] OR Slovenia[Mesh] OR Sri Lanka[Mesh] OR Somalia[Mesh] OR South Africa[Mesh] OR Sudan[Mesh] OR Suriname[Mesh] OR Swaziland[Mesh] OR Syria[Mesh] OR Tajikistan[Mesh] OR Tanzania[Mesh] OR Thailand[Mesh] OR Togo[Mesh] OR Tonga[Mesh] OR "Trinidad and Tobago"[Mesh] OR Tunisia[Mesh] OR Turkey[Mesh] OR Turkmenistan[Mesh] OR Uganda[Mesh] OR Ukraine[Mesh] OR Uruguay[Mesh] OR USSR[Mesh] OR Uzbekistan[Mesh] OR Vanuatu[Mesh] OR Venezuela[Mesh] OR Vietnam[Mesh] OR Yemen[Mesh] OR Yugoslavia[Mesh] OR Zambia[Mesh] OR Zimbabwe[Mesh] OR "Southern African Development Community"[all fields] OR "East African Community"[all fields] OR "West African Health Organisation"[all fields] OR "Sub Saharan Africa"[all fields] OR "SubSaharan Africa "[all fields] OR Bahrain[all fields] OR Manama[all fields] OR “Brunei”[all fields] OR Guyana[all fields] OR Kuwait[all fields] OR “North Korea”[all fields] OR Oman[all fields] OR Qatar[all fields] OR Doha[all fields] OR “Russian Federation”[all fields] OR “Saudi Arabia”[all fields] OR “United Arab Emirates”[all fields]
