# Supplementary material for: Alarming findings of psycho-socio-spiritual interventions on physical, mental, and social health for children with cancer and their families in low- and middle-income countries: a meta-analysis
Source: Front Psychiatry. 2025 Apr 28;16:1533599. doi: 10.3389/fpsyt.2025.1533599 (PMC12068859; doi:10.3389/fpsyt.2025.1533599)
Supplement: Supplementary Figure 1 — Comparison between the observed (blue curve) and true effect (red curve) sizes assuming a normal distribution of the population. The prediction interval that reflects the heterogeneity of the true effect showed a wide dispersion of effect size. [file SupplementaryFile1.zip › Appendix C.ZIP!/Appendix C Simulation model.htm]

model screening - Fit Least Squares
